# Supplementary material for: Molecular Epidemiology of Fosfomycin Resistant E. coli from a Pigeon Farm in China
Source: Antibiotics (Basel). 2021 Jun 25;10(7):777. doi: 10.3390/antibiotics10070777 (PMC8300711; doi:10.3390/antibiotics10070777)
Supplement: Supplementary file 1 [file antibiotics-10-00777-s001.zip › antibiotics-1250568-supplementary.pdf]

Supplementary Materials

Article

Molecular Epidemiology of Fosfomycin Resistant *E. coli* from a Pigeon Farm in China

Lu Han [1,2,†], Xiao-Qing Lu [1,2,†], Xu-Wei Liu [1,2], Mei-Na Liao [1,2], Ruan-Yang Sun [1,2], Yao Xie [1,2], Xiao-Ping Liao [1,2,3], Ya-Hong Liu [1,2,3], Jian Sun [1,2,3] and Rong-Min Zhang [1,2,3,*]

[1] National Risk Assessment Laboratory for Antimicrobial Resistance of Animal Original Bacteria, College of Veterinary Medicine, South China Agricultural University, Guangzhou 510642, P. R. China; luhan@stu.scau.edu.cn (L.H.); lxq@stu.scau.edu.cn (X.-Q.L.); xwliu11@stu.scau.edu.cn (X.-W.L.); lmn@stu.scau.edu.cn (M.-N.L.); sunruanyang@163.com (R.-Y.S.); 2017zrm@stu.scau.edu.cn (Y.X.); xpliao@scau.edu.cn (X.-P.L.); lyh@scau.edu.cn (Y.-H.L.); jiansun@scau.edu.cn (J.S.)

[2] Guangdong Provincial Key Laboratory of Veterinary Pharmaceutics Development and Safety Evaluation, College of Veterinary Medicine, South China Agricultural University, Guangzhou 510642, P. R. China

[3] Guangdong Laboratory for Lingnan Modern Agriculture, South China Agricultural University, Guangzhou, China

* Correspondence: zrm@scau.edu.cn

† These authors contributed equally to this work.

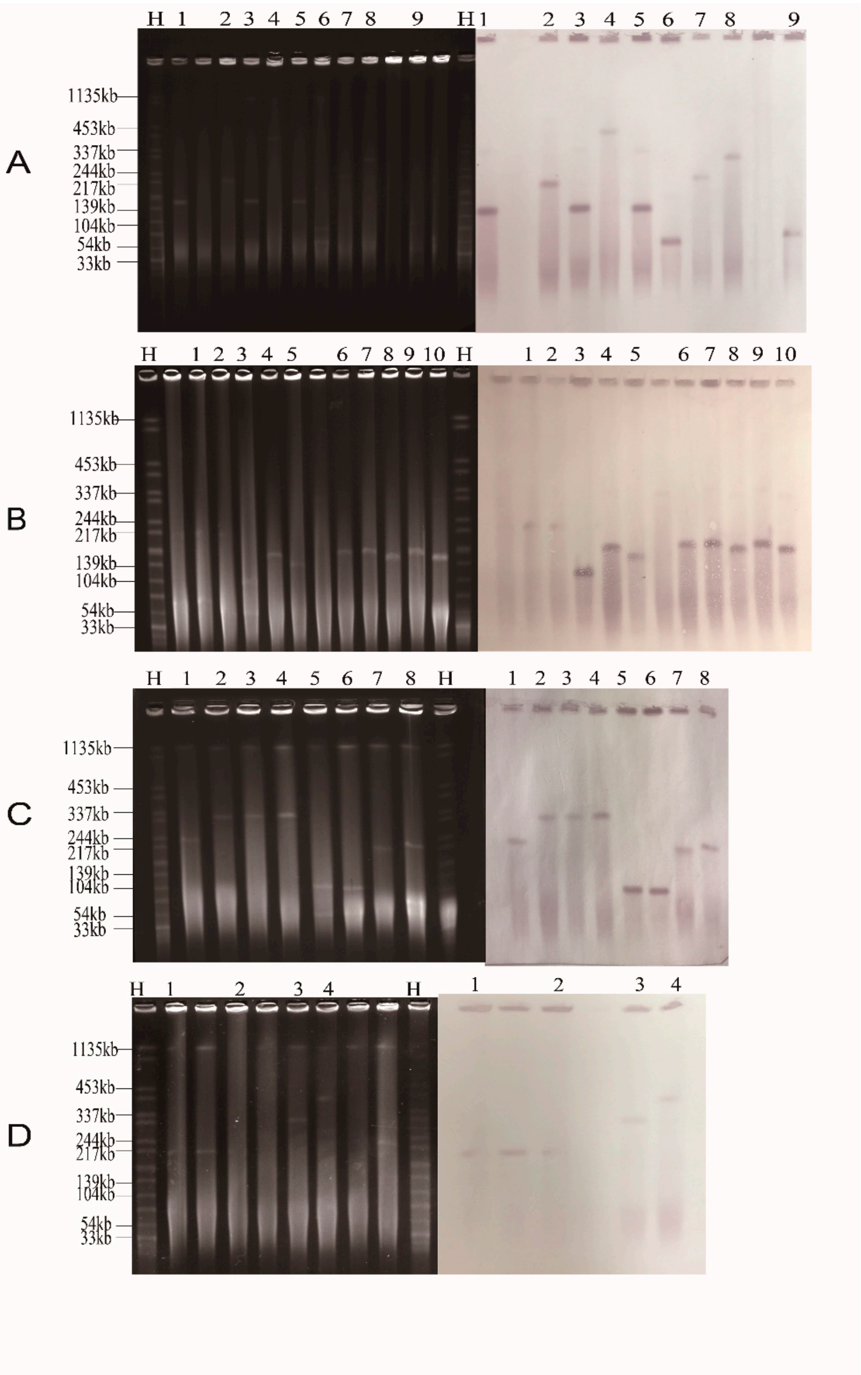

Figure S1. S1-nuclease PFGE mapping and Southern blot analysis of *fosA3*-carrying plasmids on different sizes. A. Strains present in lanes 1 to 9 were B4, E9-1, M6-1, W1-1, B20-2-2, W3-1, Y1-1-1, E15-1, and M1-2, respectively. B. Strains present in Lanes 1 to 10 were E3, W6-2-1, M5-1, B20-1-1, B20-1-2, E4-1, E5, E9-2, W6-2-2, and E4-2-2, respectively. C. Strains presenting Lanes 1 to 8 were X2-3, W2-1, E19-2, E13-2, B17-1-2, E11- 2, B15-2 and E2, respectively. D. Strains present in Lanes 1 to 4 were E6-1, W6-2-1, M4-1, and M1-1, respectively. H, H9812 marker.

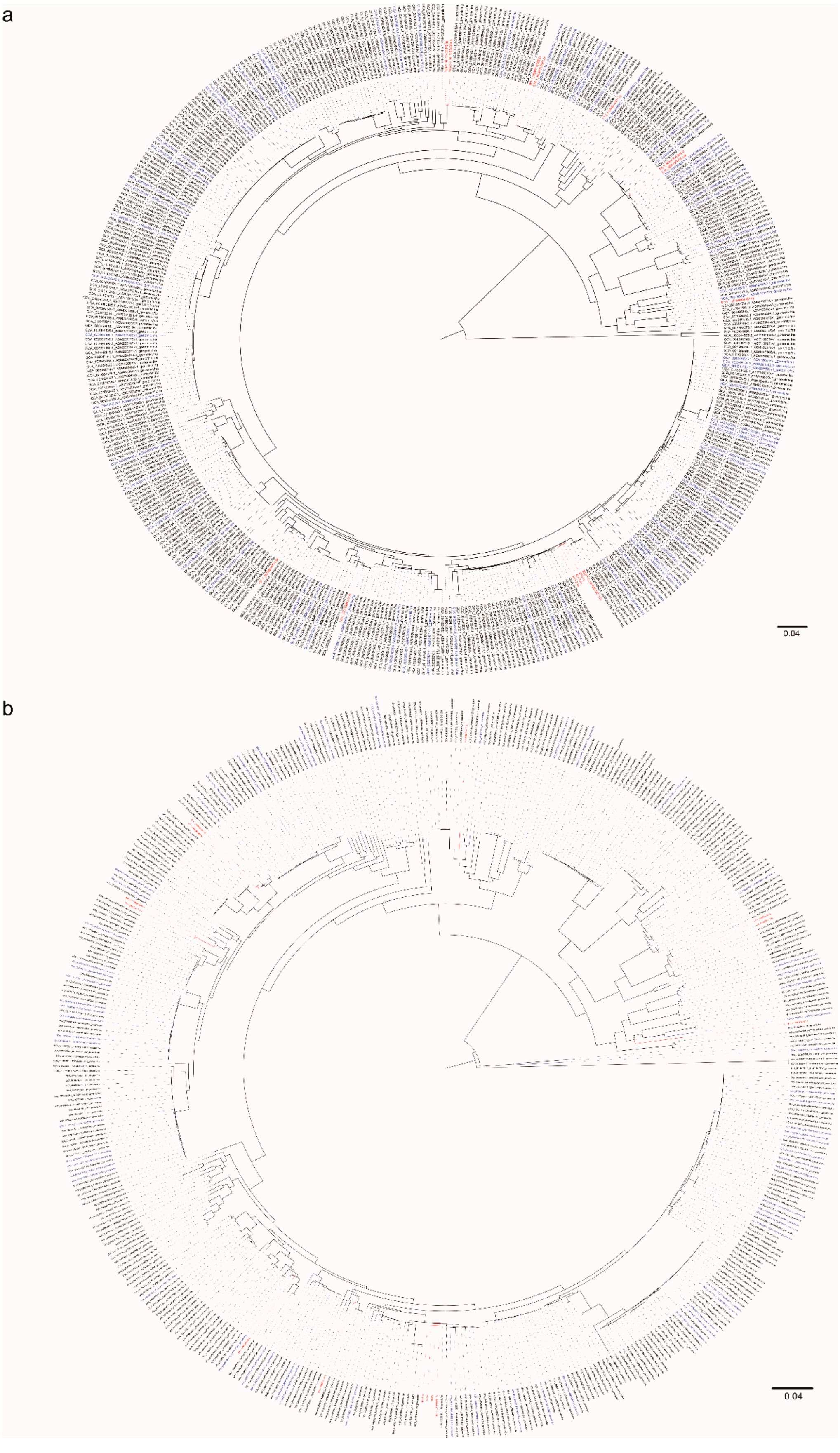

Figure S2. Phylogenetic analysis of the *fosA3* positive *E. coli* from public repository and current study. The 915 *fosA3* positive *E. coli* from public repository were divided into two groups randomly and each group were employed to constructed a phylogenetic tree with the 14 isolates in current study (a and b), respectively. The red clades represent the *fosA3* positive *E. coli* in this study and the blue clades represent the selected 130 representative isolates.

Table S1. Sample collection, strain identification and MIC results.

Sample	Source	Gene	Isolate	CHL	SXT	AMP	CTX	CIP	MEM	TET	AMK	FFC	FOS	TIG	Antibiotic resistance spectrum
B4	Pigeon feces	fosA3	*E. coli*	1	2.5	>256	8	64	0.03	64	2	8	>256	2	FOS CTX TET CIP AMP CS
B12	Pigeon feces	fosA3	*E. coli*	1	160	>256	8	16	0.03	64	4	4	>256	2	FOS CTX TET CIP AMP S/T CS
B14-1	Pigeon feces	fosA3	*E. coli*	1	160	256	4	64	0.015	64	0.5	64	>256	2	FOS CTX TET CIP AMP S/T FFC GEN CS
B14-2	Pigeon feces	fosA3	*E. coli*	1	160	128	4	64	0.015	64	0.5	128	>256	2	FOS CTX TET CIP AMP S/T FFC CS
E7-4	Pigeon feces	fosA3	*E. coli*	1	160	>256	32	256	0.03	128	2	128	>256	2	FOS CTX TET CIP AMP S/T FFC CS
E8-1	Pigeon feces	fosA3	*E. coli*	1	160	>256	16	256	0.03	128	1	128	>256	2	FOS CTX TET CIP AMP S/T FFC CS
E8-2	Pigeon feces	fosA3	*E.coli*	1	160	>256	16	128	0.03	128	2	128	>256	4	FOS CTX TET CIP AMP S/T FFC CS
E9-1	Pigeon feces	fosA3	*E. coli*	1	160	>256	8	16	0.03	64	4	16	>256	8	FOS CTX TET CIP AMP S/T CS
M6-1	cecum contents	fosA3	*E. coli*	1	160	>256	8	16	0.03	64	4	8	>256	2	FOS CTX TET CIP AMP S/T CS
M6-2	cecum contents	fosA3	*E. coli*	1	160	>256	8	16	0.03	64	4	8	>256	2	FOS CTX TET CIP AMP S/T CS
W1-1	sewage	fosA3	*E. coli*	1	160	>256	4	64	0.015	64	1	8	>256	2	FOS CTX TET CIP AMP S/T GEN CS
W1-2	sewage														
M7	cecum contents		*P. mirabilis*												
M8	cecum contents		*Proteus mirabilis*												
B19	Pigeon feces	fosA3	*E. coli*	1	160	>256	4	32	0.015	64	2	8	>256	4	FOS CTX TET CIP AMP S/T CS
M5-1	cecum contents	fosA3	*E. coli*	1	5	>256	64	16	0.03	128	2	8	>256	1	FOS CTX TET CIP AMP CS
B20-1-1	Pigeon feces	fosA3	*E. coli*	1	5	>256	4	64	0.015	64	2	8	>256	2	FOS CTX TET CIP AMP CS
B20-1-2	Pigeon feces	fosA3	*E. coli*	4	160	>256	8	16	0.015	64	4	16	>256	4	FOS CTX TET CIP AMP S/T CS
B20-2-1	Pigeon feces		*C. freundii*												
B20-2-2	Pigeon feces	fosA3	*E. coli*	1	2.5	>256	16	64	0.015	128	2	4	>256	4	FOS CTX TET CIP AMP CS
W2-1	sewage	fosA3	*E. coli*	1	160	>256	4	64	0.03	64	1	2	>256	2	FOS CTX TET CIP AMP S/T GEN CS
W3-1	sewage	fosA3	*E. coli*	1	80	>256	4	64	0.03	64	2	8	>256	2	FOS CTX TET CIP AMP CS
W4-1	sewage		*P. mirabilis*												
W5-1	sewage														
W2-2-1	sewage	fosA3	*E. coli*	1	160	>256	16	256	0.03	128	2	64	>256	2	FOS CTX TET CIP AMP S/T FFC GEN CS
W2-2-2	sewage		*P. mirabilis*												
W5-2-1	sewage		*P. mirabilis*												
W5-2-2	sewage		*P. mirabilis*												
W6-1	sewage	fosA3	*E. coli*	1	160	>256	4	256	0.03	64	1	64	>256	1	FOS CTX TET CIP AMP S/T FFC CS
W7-1	sewage														
W9-1	sewage														

W9-2	sewage		*Proteus mirabilis*												
X1-1	liquor pericardii														
X2-3	liquor pericardii	fosA3	*E. coli*	1	160	128	16	16	0.03	128	2	4	>256	2	FOS CTX TET CIP AMP S/T CS
X4-2	liquor pericardii		*P. mirabilis*												
X5-1	liquor pericardii		*E. coli*												
X1-3-1	liquor pericardii		*E. coli*												
X1-3-2	liquor pericardii		*P. mirabilis*												
Y1-1-1	flies	fosA3	*E. coli*	1	160	128	16	16	0.03	128	2	4	>256	2	FOS CTX TET CIP AMP S/T
Y1-1-2	flies														
Y1-2	flies														
Y1-3	flies		*P. mirabilis*												
Y2-1	flies														
Y2-2	flies		*C. freundii*												
M4-1	cecum contents	fosA3	*E. coli*	1	160	>256	16	16	0.03	64	4	8	>256	2	FOS CTX TET CIP AMP S/T CS
M4-2	cecum contents	fosA3	*E. coli*	1	160	>256	16	16	0.03	32	4	8	>256	2	FOS CTX TET CIP AMP S/T CS
E15-1	Pigeon feces	fosA3	*E. coli*	1	160	>256	2	64	0.015	64	1	8	>256	2	FOS CTX TET CIP AMP S/T GEN CS
E15-2	Pigeon feces	fosA3	*E. coli*	1	160	>256	8	16	0.03	32	2	4	>256	2	FOS CTX TET CIP AMP S/T CS
M1-1	cecum contents	fosA3	*E. coli*	1	160	>256	4	64	0.015	128	1	4	>256	2	FOS CTX TET CIP AMP S/T GEN CS
M1-2	cecum contents	fosA3	*E. coli*	1	20	>256	32	16	0.03	128	2	4	>256	1	FOS CTX TET CIP AMP CS
B17-1-1	Pigeon feces		*P. mirabilis*												
B17-1-2	Pigeon feces	fosA3	*E. coli*	1	20	>256	32	16	0.03	128	2	2	>256	1	FOS CTX TET CIP AMP CS
E16	Pigeon feces	fosA3	*E. coli*	1	160	>256	16	128	0.03	64	2	128	>256	2	FOS CTX TET CIP AMP S/T FFC CS
E19-2	Pigeon feces	fosA3	*E. coli*	1	160	>256	8	64	0.015	64	1	32	>256	2	FOS CTX TET CIP AMP S/T FFC GEN CS
B15-2	Pigeon feces	fosA3	*E. coli*	1	160	>256	8	16	0.03	32	2	8	>256	2	FOS CTX TET CIP AMP S/T CS
E2	Pigeon feces	fosA3	*E. coli*	1	160	>256	8	256	0.03	64	1	128	>256	1	FOS CTX TET CIP AMP S/T FFC CS
X4-3	cecum contents		*P. mirabilis*												
X4-4	cecum contents		*P. mirabilis*												
X5-2	cecum contents		*E. coli*												
E7-1	Pigeon feces	fosA3	*E. coli*	1	160	>256	16	256	0.03	128	1	128	>256	2	FOS CTX TET CIP AMP S/T FFC CS

E7-2	Pigeon feces	fosA3	*E. coli*	1	160	>256	16	128	0.03	64	2	128	>256	2	FOS CTX TET CIP AMP S/T FFC CS
E7-3	Pigeon feces	fosA3	*E. coli*	1	160	>256	16	128	0.03	64	2	64	>256	2	FOS CTX TET CIP AMP S/T FFC CS
E6-1	Pigeon feces	fosA3	*E. coli*	1	160	>256	4	256	0.03	128	1	128	>256	1	FOS CTX TET CIP AMP S/T FFC CS
E6-2	Pigeon feces		*P. mirabilis*												
E3	Pigeon feces	fosA3	*E. coli*	1	160	>256	8	256	0.03	64	1	128	>256	4	FOS CTX TET CIP AMP S/T FFC CS
E4-1	Pigeon feces	fosA3	*E. coli*	1	2.5	>256	8	64	0.03	64	2	4	>256	2	FOS CTX TET CIP AMP CS
E5	Pigeon feces	fosA3	*E. coli*	1	2.5	>256	8	64	0.03	64	2	2	>256	2	FOS CTX TET CIP AMP CS
E9-2	Pigeon feces	fosA3	*E. coli*	1	2.5	>256	8	64	0.03	2	2	2	>256	2	FOS CTX TET CIP AMP CS
E11-2	Pigeon feces	fosA3	*E. coli*	1	2.5	>256	64	32	0.03	128	2	4	>256	1	FOS CTX TET CIP AMP CS
E13-2	Pigeon feces	fosA3	*E. coli*	1	160	>256	4	64	0.015	128	1	4	>256	2	FOS CTX TET CIP AMP S/T GEN CS
W5-3-1	sewage	fosA3	*E. coli*	1	160	>256	8	16	0.03	64	2	4	>256	2	FOS CTX TET CIP AMP S/T FFC CS
W5-3-2	sewage	fosA3	*E. coli*	1	80	>256	8	16	0.03	64	4	8	>256	2	FOS CTX TET CIP AMP S/T CS
W6-2-1	sewage	fosA3	*E. coli*	1	2.5	>256	8	64	0.03	64	2	2	>256	2	FOS CTX TET CIP AMP S/T CS
W6-2-2	sewage	fosA3	*E. coli*	1	2.5	>256	8	64	0.03	64	2	2	>256	2	FOS CTX TET CIP AMP CS
C1-1	dust														
C1-2	dust		*Proteus mirabilis*												
C1-3	dust														
E4-2-1	Pigeon feces	fosA3	*E. coli*	8	160	>256	4	256	0.03	128	1	128	>256	1	FOS CTX TET CIP AMP S/T FFC CS
E4-2-2	Pigeon feces	fosA3	*E. coli*	16	2.5	>256	8	64	0.03	64	>256	128	>256	2	FOS CTX TET CIP AMP GEN FFC CS
